# Regulation of PTEN expression by noncoding RNAs

**DOI:** 10.1186/s13046-018-0898-9

**Published:** 2018-09-10

**Authors:** Wang Li, Ting Zhang, Lianying Guo, Lin Huang

**Affiliations:** Department of Pathophysiology, College of Basic Medical Sciences, Dalian Medical University, Dalian, Liaoning 116044 People’s Republic of China

**Keywords:** PTEN, Noncoding RNA, miRNA, lncRNA, Methylation, ceRNA

## Abstract

Phosphatase and tensin homologue (PTEN) triggers a battery of intracellular signaling pathways, especially PI3K/Akt, playing important roles in the pathogenesis of multiple diseases, such as cancer, neurodevelopmental disorders, cardiovascular dysfunction and so on. Therefore PTEN might be a biomarker for various diseases, and targeting the abnormal expression level of PTEN is anticipated to offer novel therapeutic avenues. Recently, noncoding RNAs (ncRNAs) have been reported to regulate protein expression, and it is definite that PTEN expression is controlled by ncRNAs epigenetically or posttranscriptionally as well. Herein, we provide a review on current understandings of the regulation of PTEN by ncRNAs, which could contribute to the development of novel approaches to the diseases with abnormal expression of PTEN.

## Background

**Fig. 1:**
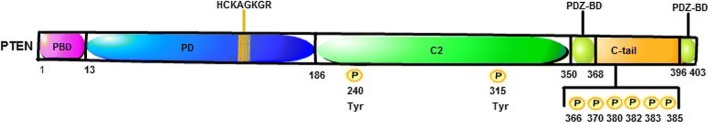
The Structure of PTEN. PTEN encodes a 403-amino acid peptide, which is composed of a phosphatidylinositol-4, 5-bisphosphate-binding domain (PBD) (residues 1–13), a catalytic phosphatase domain (PD) (residues 14–185), a C2 membrane binding domain (C2D)(residues 186–350), and a C-terminal tail (residues 351–403). The PD includes a conserved catalytic motif HCKAGKGR. The C2 domain includes two tyrosine phosphorylation sites (Y240 and Y315). There are two PDZ-binding domains (PDZ-BD) and six phosphorylation sites in the C-terminal tail. PDZ, post-synaptic density protein (PSD95), Drosophila discs large (Dlg) and the tight junction protein zonula occludens-1 (ZO-1)

*Phosphatase and tensin homologue (PTEN)*, also named as *MMAC1* (mutated in multiple advanced cancers), is located on chromosome 10q23.31 [1, 2]. *PTEN* encodes a 403-amino acid peptide, which is composed of a phosphatidylinositol-4,5-bisphosphate-binding domain (PBD) (residues 1–13), a catalytic phosphatase domain (PD) (residues 14–185), a C2 membrane binding domain (C2D)(residues 186–350), and a C-terminal tail (residues 351–403) [3, 4]. The PD includes a conserved catalytic motif HCKAGKGR, contributing to the dual lipid and protein phosphatase activity of PTEN [4, 5]. The C2 domain includes two tyrosine phosphorylation sites (Y240 and Y315). The PDZ-binding domain (post-synaptic density protein (PSD95), Drosophila discs large (Dlg) and the tight junction protein zonula occludens-1 (ZO-1)) associates with the phosphatase activity, membrane association and stability of PTEN. There are two PDZ-binding domains and six phosphorylation sites in the C-terminal tail, including threonine 366 (Thr366), serine 370 (Ser370), Ser380, Thr382, Thr383 and Ser385 [6–11] (Fig. 1).

**Fig. 2:**
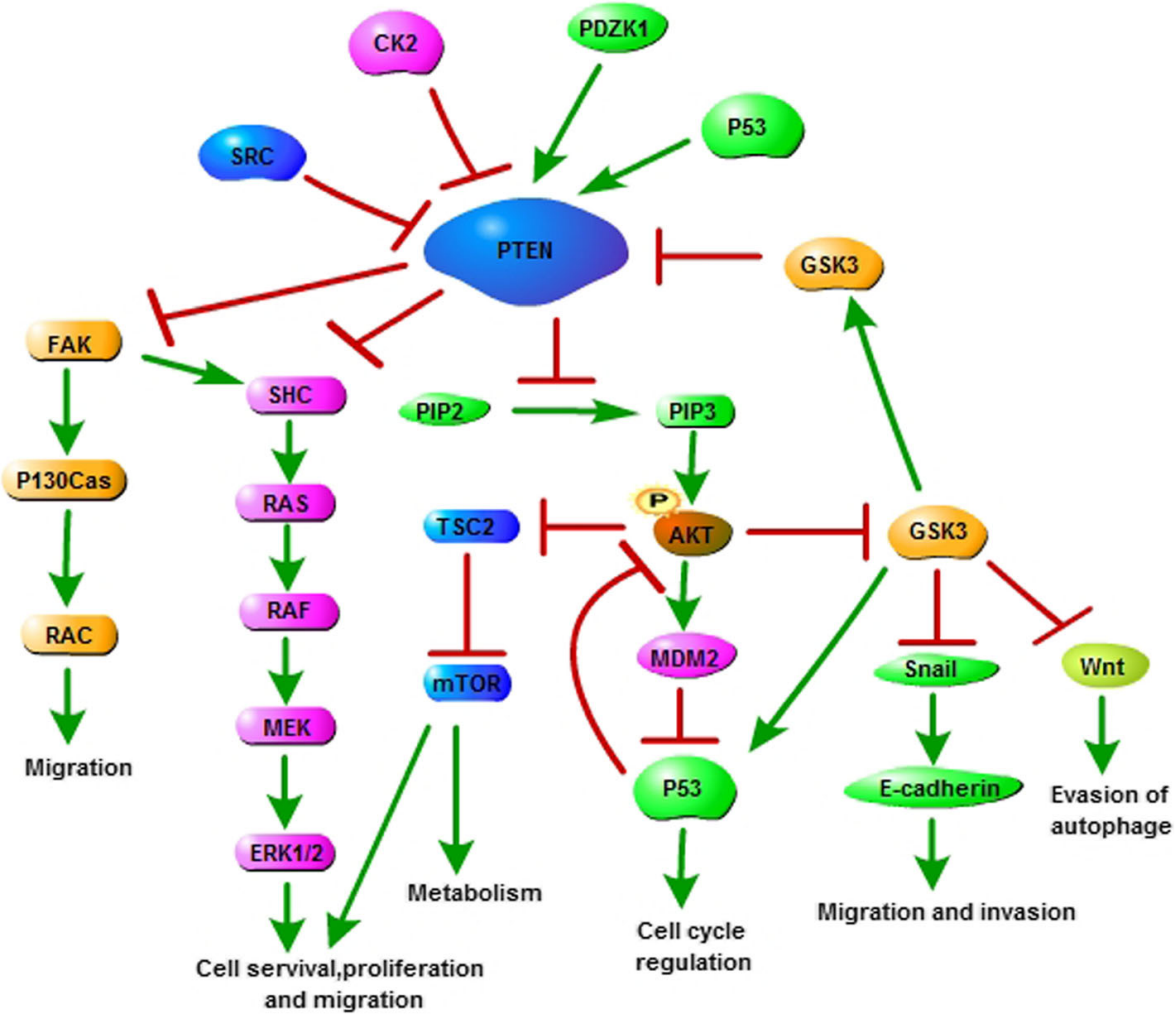
The schematic representation of the major signaling pathways in which PTEN is involved. CK2, casein kinase II; PDZK1, PDZ-containing 1; GSK3, Glycogen synthase kinase3; FAK, Focal adhesion kinase; Rac, Ras-related C3 botulinum toxin substrate; SHC, Src homology 2-containing protein; MEK, MAPKK(mitogen-activated protein kinase kinase); ERK1/2, Extracellular signal-related kinase 1/2; PIP3, Phosphatidylinositol (3,4,5)-trisphosphate (PtdIns(3,4,5)*P*_3_); Akt, Protein kinase B (PKB); MDM2, Mouse double minute 2 homolog; TSC2, Tuberous Sclerosis Complex 2; mTORC, Mammalian target of rapamycin complex; CSCs, Cancer stem cells

PTEN contributes to the control of several important cellular signaling pathways. PTEN dephosphorylates phosphatidylinositol (3,4,5)-triphosphate (PIP3), therefore represses the activation of phosphatidylinositol-3-kinase (PI3K)/Akt and the mammalian target of rapamycin (mTOR) signaling pathway, Akt/ glycogen synthase kinase3(GSK-3)/Snail signaling pathway, or Akt/GSK-3/Wnt/ signaling pathway. Furthermore, GSK-3 interacts with and phosphorylates PTEN, which contributes to the inactivation of PTEN. Focal adhesion kinase (FAK) is dephosphorylated by PTEN directly, leading to the inactivation of FAK/p130Cas pathway. PTEN also dephosphorylates Src homology 2-containing protein (Shc) directly, and inhibits the activation of Shc/Raf/ERK1/2 (extracellular signal-regulated kinase) signaling cascade. Through controlling these pathways, PTEN ultimately represses cell survival, proliferation, metastasis and so on [12–18] (Fig. 2).

PTEN expression alteration is crucial to the pathogenesis of cancer and other diseases. Low level of PTEN caused by homozygous deletions, frameshift, nonsense mutations or hypermethylation of the gene or destability of the protein occurs frequently in various human cancers [19–23] and *PTEN* depletion in mice leads to a substantial rise in tumorigenesis [24, 25]. *PTEN* mutations were reported as a cause of obesity and autism spectrum disorders [26–28]. PTEN protein level was decreased in an OVA-induced-asthma mouse model, and the administration of PTEN expressing adenovirus remarkably reduced bronchial inflammation and airway hyperresponsiveness [29]. However, high level of PTEN either contributes to pathological processes. Elevated PTEN expression was observed in endothelium of atherosclerotic brachial arteries from hemodialysis patients. PTEN overexpression stimulated the thrombosis formation of arteriovenous graft by inducing endothelial dysfunction [30]. PTEN negatively regulates neuron survival, and PTEN downregulation showed neuroprotective effects in mouse models of neuron death and Parkinson’s disease [31, 32]. Inhibition of PTEN rescued synaptic function and cognition in cellular and animal models of Alzheimer’s disease, whereas *PTEN* transgenic mice displayed synaptic depression [33]. In brief, abnormal PTEN expression level is associated to multiple diseases. Understanding the regulation mechanisms of PTEN expression and maintaining the homeostasis of PTEN should be beneficial.

**Fig. 3:**
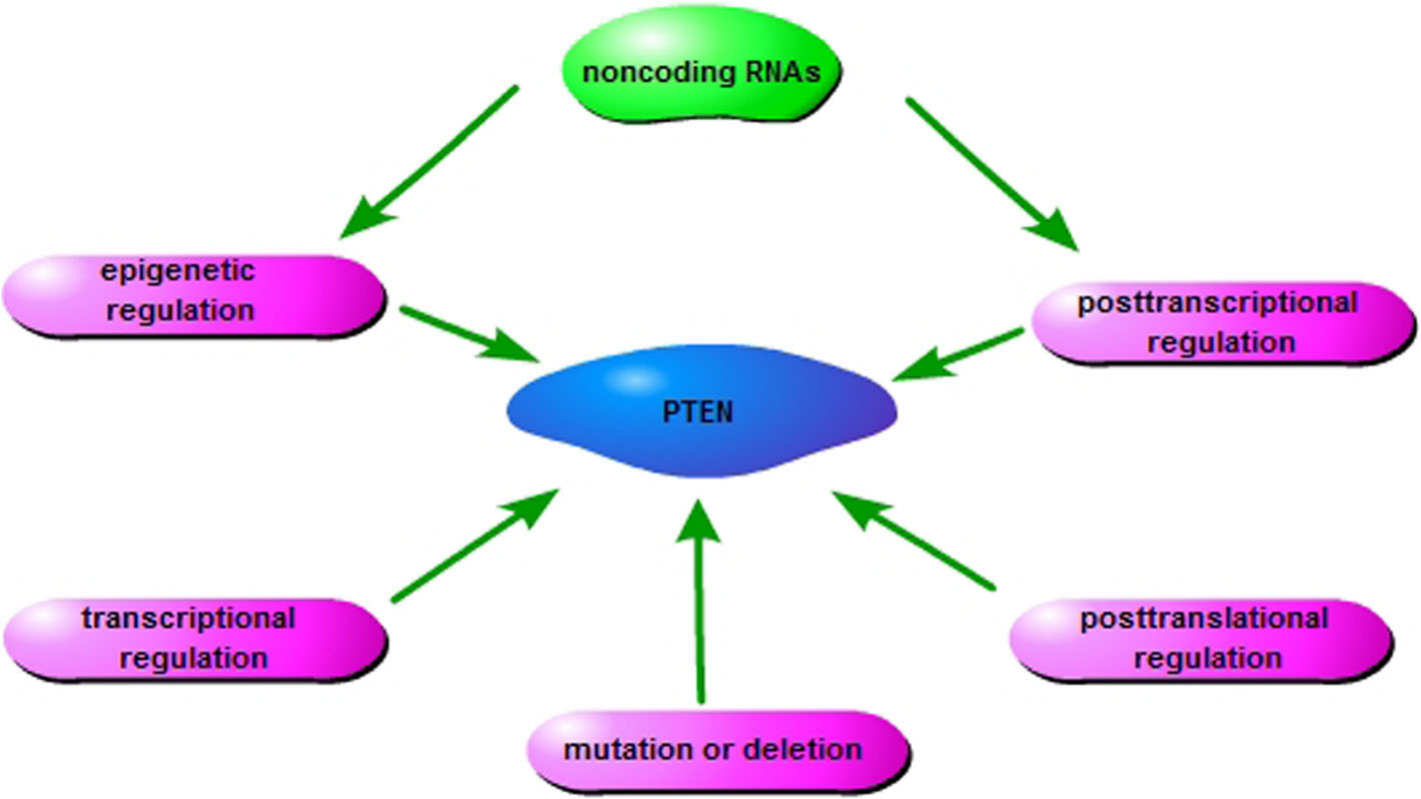
The regulation of PTEN expression. PTEN expression is dynamically regulated by various events, including genomic mutation or deletion, transcriptional, epigenetic, posttranscriptional, and posttranslational modulation. Noncoding RNAs epigenetically or posttranscriptionally regulate PTEN expression

The expression and activity of PTEN is modulated by several upstream molecules. P53 binds *PTEN* promoter and induces its transcription [34]. PDZK1 (PDZ-containing 1) induces PTEN dephosphorylation through binding the PDZ-binding domain in the PTEN C-terminal domain, which promotes the anti-oncogenic function of PTEN. Protein kinase CK2 (formerly casein kinase II) interacts with and phosphorylates PTEN C-terminal tail, which contributes to maintain PTEN stability [35]. Src inhibits PTEN activity to promote the post-ischaemic contractile recovery in apelin-induced cardioprotection [36]. Recently, with the development of the study on noncoding RNAs (ncRNAs), the control of PTEN expression by ncRNAs attracted more attention. Herein, we focus on the regulation of PTEN expression by ncRNAs, which is supposed to provide a reference for the coming laboratory and clinical studies on PTEN regulation (Fig. 3).

More than 98% DNAs that do not encode proteins are called ncRNAs [37, 38]. In general, ncRNAs are classified into two groups as small ncRNAs (≤ 200 nt) and long ncRNAs (lncRNAs) (> 200 nt). MicroRNAs (miRNAs) (~ 18 to 24 nt) are an important group of small ncRNAs, which epigenetically or posttranscriptionally control the expression of the target mRNAs by pairing to them, leading to the alteration of transcription, mRNA stability or translation [39–42]. LncRNAs take up a great proportion in the “transcriptome”, which play vital gene regulatory roles in the chromatin modification, transcriptional regulation, posttranscriptional regulation and so on [43, 44]. Emerging evidence indicates that PTEN functions in a dosage-dependent manner during tumor development [24, 45]. NcRNAs are key regulators of PTEN dosage, including miRNAs and lncRNAs, which delicately modulate the PTEN expression (Tables 1 and 2).

**Table 1:** Regulation of PTEN expression by miRNAs

MiRNA	PTEN expression	Mechanism	Disease	Reference
miR-21	Down	directly targeting PTEN mRNA	Gastric cancer, HNSCC, CCRCC	[52–54]
miR-130	Down	directly targeting PTEN mRNA	Bladder cancer, Breast invasive carcinoma, HCAECs injury, Inflammatory responses, PD, Lung adenocarcinoma, Colon adenocarcinoma	[55–58]
miR-130	Up	directly targeting PTEN mRNA	NSCLC	[59]
miR-451	Up	directly targeting PTEN mRNA	Lung cancer, Ovarian cancer	[60, 61]
miR-221 /222	Down	directly targeting PTEN mRNA	NSCLC,HCC, TRAIL-induced cell death	[62]
miR-301a	Down	directly targeting PTEN mRNA	Breast cancer, Neuronal death, Ewing’s carcoma, Melanoma, Insulin resistance	[63–67]
miR-214	Down	directly targeting PTEN mRNA	Tumorigenesis, Immunology, Cardiac injury	[68–71]
miR-494	Down	directly targeting PTEN mRNA	Ischemia/Reperfusion -induced myocardial injury	[72, 73]
miR-155-5p/130b/616/19/92a/10a/106a/429/26a /486-5p	Down	directly targeting PTEN mRNA	HCC, NSCLC, Breast cancer, Lung cancer, Colorectal Cancer, Chronic myeloid leukemia, Intestinal cancer, Acute T-cell lymphoblastic leukemia	[74–84]
miR-29	Up	inducing the hypomethylation of PTEN promoter by inhibiting DNMT1, DNMT3b and SET1A expression	Liver fibrosis	[87, 88]
miR-101	Up	inducing the hypomethylation of PTEN promoter by inhibiting DNMT3A expression	Lung cancer	[89, 90]
miR-185	Up	inducing the hypomethylation of PTEN promoter by inhibiting DNMT1 expression	HCC	[91]

**Table 2:** Regulation of PTEN expression by lncRNAs

LncRNA	PTEN expression	Mechanism	MiRNA	Disease	Reference
PTENP1	Up	acting as ceRNAs	miR-21, miR-17, miR-214, miR-19, miR-20, miR-93, miR-106b, miR-26	CCRCC, OSCC, HCC, Gastric cancer	[54, 96–101]
HOTAIR	Up	acting as ceRNAs	miR-19	Cardiac hypertrophy	[105]
Linc-USP16	Up	acting as ceRNAs	miR-21,miR-590-5p	HCC	[106]
LncRNA-BGL3	Up	acting as ceRNAs	miR-17, miR-20a, miR-20b, miR-93, miR-106a	Chronic myeloid leukemia	[80]
CASC2	Up	acting as both ceRNAs and downregulators of miRNAs	miR-21, miR-181a	Osteosarcoma, Glioma, Cervical cancer	[107–109]
MEG3	Up	acting as both ceRNAs and downregulators of miRNAs	miR-1297, miR-19a, miR-21	Breast cancer, Glioma, CAD	[111–113]
lncRNA GAS5	Up	acting as both ceRNAs and downregulators of miRNAs	miR-21, miR-103, miR-196a, miR-205, miR-32-5p	HER2-positive breast cancer, HCC, NSCLC, Cardiac fibrosis, Endometrial cancer, Cervical cancer, Pancreatic cancer	[114–120]
XIST	Up	acting as both ceRNAs and downregulators of miRNAs	miR-181a, MiR-494	HCC, Spinal Cord Injury	[121, 122]
NBAT1	Up	acting as both ceRNAs and downregulators of miRNAs	miR-21	Osteosarcoma	[123]
lnc-2 /lnc-6	Up	acting as both ceRNAs and downregulators of miRNAs	miR-26a	Prostate cancer, Glioma	[126, 127]
FER1L4	Up	acting as both ceRNAs and downregulators of miRNAs	miR-106a-5p	Colon cancer, Gastric cancer	[130, 131]
lincRNA-p21	Up	acting as both ceRNAs and downregulators of miRNAs	miR-181b	Liver fibrosis	[132]
PTENP1 asRNA β	Up	increasing the stability and miRNA sponge activity of PTENP1	–	–	[133]
HOTAIR	Down	enhancing PTEN methylation via miRNA sponging	miR-29b	Liver Fibrosis, LSCC	[134, 135]
PTENP1 asRNA α	Down	enhancing PTEN methylation via the recruitment of DNMT3a and EZH2	–	–	[133]

## MiRNAs modulate PTEN expression

### Altering PTEN expression by directly targeting PTEN mRNA

MiR-21 is one of the first identified mammalian microRNAs. The human *miR-21* gene is located at chromosome 17q23.2 within a coding gene *TMEM49* (also called vacuole membrane protein), which is highly conserved [46]. Early lineage tracing studies demonstrated that miR-21 was upregulated in various diseases, including acute pancreatitis [47], Myelodysplastic syndromes [48], severe steroid-insensitive allergic airway disease [49], liver cancer [50] and lung cancer [51].

**Fig. 4:**
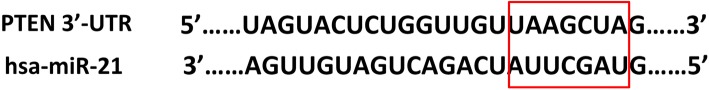
A Predicted miR-21 binding site within the 3’UTR of PTEN mRNA. By Target Scan Human Release 7.0 (http://www.targetscan.org)

PTEN is one of the important targets negatively regulated by miR-21. The 3′UTR of human PTEN contains a putative region that is able to pair to the seed sequence of miR-21 (Fig. 4). The exosomal miR-21 transferred from macrophages downregulated the PTEN level in gastric cancer cells, which resulted in the suppression of cell apoptosis and activation of PI3K/AKT signaling pathway [52]. Inhibition of miR-21 reversed EMT by increasing PTEN protein level in head and neck squamous cell carcinoma (HNSCC), resulting in the suppression of cell proliferation and motility [53]. MiR-21 was able to directly target the 3′UTR of PTEN, increasing clear-cell renal cell carcinoma (CCRCC) cell migration, invasion both in vitro and in vivo [54].

Expression of miR-130 family members has been recently reported to be correlated inversely to PTEN expression in bladder cancer, breast invasive carcinoma, lung adenocarcinoma and colon adenocarcinoma [55, 56]. Overexpression of miR-130a increased cell proliferation and motility via repression of PTEN expression, associated with the upregulation of FAK and Akt phosphorylation [55–57]. MiR-130a decreases the PTEN level to activate PI3K/Akt/eNOS (endothelial nitric oxide synthase) signaling pathway, promoting human coronary artery endothelial cells (HCAECs) injury and inflammatory responses [57]. Exogenous expression of miR-130a prevented midbrain dopaminergic (mDA) neuron degeneration in Parkinson’s disease (PD) by suppressing the synthesis of PTEN [58].

Controversially, miR-130 was also found to be downregulated and positively correlated to PTEN levels in non-small cell lung cancer (NSCLC) tissue samples. The upregulation of miR-130 significantly increased PTEN expression, inhibited NSCLC cell growth and enhanced cell apoptosis both in *vitro* and in *vivo* [59]. Even the same pairing sequence of miR-130 and PTEN 3’UTR were used, opposite results were obtained in dual luciferase reporter assays from two reports. The relative activity of the luciferase harboring PTEN 3’UTR was promoted in A549 cells but repressed in 293 T cells by miR-130 [56, 59]. Although the mechanisms remain obscure, a tissue-specific pattern is possible for the regulation of PTEN by miR-130. MiR-130 might regulate PTEN expression through different ways according to the cellular context. PTEN protein was found to be slightly increased after the pre-miR-451-transfection in lung cancer cells [60]. Both miR-451 and PTEN expression level was reported to be significantly reduced in ovarian cancer [61].

Over the past decade, mountains of results show that the interaction of PTEN with miRNAs related to different diseases. MiR-221 and miR-222 were reported to be upregulated in aggressive NSCLC and hepatocarcinoma (HCC) cells, and conferred resistance to TNF-related apoptosis-inducing ligand (TRAIL)-induced cell death by targeting PTEN [62]. MiR-301a mediates the tumorigenesis of breast cancer, Ewing’s carcoma and melanoma, prevents neuronal death, and contributes to insulin resistance via decreasing PTEN protein level [63–67]. MiR-214 induces tumorigenesis, stimulates immunology, and protects cardiac injury through inhibiting PTEN expression [68–71]. MiR-494 targets PTEN and activates Akt pathway, leading to protect against ischemia/reperfusion -induced myocardial injury [72, 73]. There are also many other miRNAs directly targeting PTEN, such as miR-155-5p [74], miR-130b [75], miR-616 [76], miR-19 [77], miR-92a [78], miR-10a [79], miR-106a [80], miR-429 [81], miR-26a [82, 83] and miR-486-5p [84]. Consistent with miR-21, these miRNAs directly bind to the 3′UTR of human PTEN, and inhibit PTEN expression.

### Upregulating PTEN expression by targeting DNA methyltransferases (DNMTs)

**Fig. 5:**
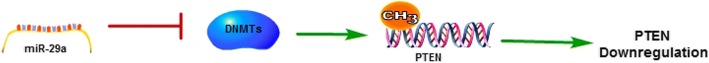
MiR-29a upregulates PTEN expression by targeting DNMTs. MiR-29a could repress DNMTs at posttranscriptional level, resulting in a decrease of CpG island methylation of the PTEN promoter. DNMTs, DNA methyltransferases

DNA methyltransferases (DNMTs) are the enzymes for DNA methylation, transferring a methyl group to the cytosine residues of DNA. DNA methylation of a gene promoter typically represses the gene transcription. The promoter region of the *PTEN* gene consists of three methylation sites. Overexpression of DNMT1 led to PTEN downregulation due to the CpG island methylation in promoter, which promoted tumorigenesis of breast cancer, ovarian cancer and acute myeloid leukemia (AML) [85, 86]. MiRNAs targeting DNMTs increase the PTEN expression. MiR-29a was found to inhibit DNMT1, DNMT3b and SET domain containing 1A (SET1A) expression, resulting in elevated PTEN expression and decreased offibrogenic activities in hepatic stellate cells (HSCs) [87]. Curcumin treatment suppressed liver fibrosis by inducing miR-29b expression in HSCs, which led to the low expression of DNMT3b and PTEN hypomethylation [88] (Fig. 5). Bioinformatics and dual luciferase reporter assays demonstrated that DNMT3A is a target of miR-101 [89]. Introduction of miR-101 inhibitor increased the protein level of DNMT3A instead of the mRNA expression. Overexpression of miR-101 or silencing of DNMT3A induced the hypomethylation of *PTEN* promoter which was verified by a methylation specific PCR assay [90]. The expression of miR-185 was inhibited in cultured human HCC cells [91]. Introduction of miR-185 mimics significantly reduced DNMT1 expression, decreased *PTEN* promoter methylation and increased the protein level of PTEN. MiR-185 overexpression decreased the reporter activity of the luciferase with DNMT1 3’UTR, and forced expression of DNMT1 reversed the loss of *PTEN* promoter methylation mediated by miR-185.

## LncRNAs modulate PTEN expression indirectly

LncRNAs have multiple important functions in cellular and developmental processes. LncRNAs may carry out both gene inhibition and activation through diverse mechanisms [43, 44]. The studies on the lncRNAs associated with PTEN suggest that lncRNAs modulate PTEN expression by altering either the related miRNAs or promoter methylation.

### Acting as competing endogenous RNAs (ceRNAs)

LncRNAs can act as competing endogenous RNAs (ceRNAs) to indirectly regulate mRNAs through the shared miRNAs. LncRNAs compete the seed sites of miRNAs with their target mRNAs, leading to block the effects of miRNAs on the mRNA targets [92–95].

**Fig. 6:**
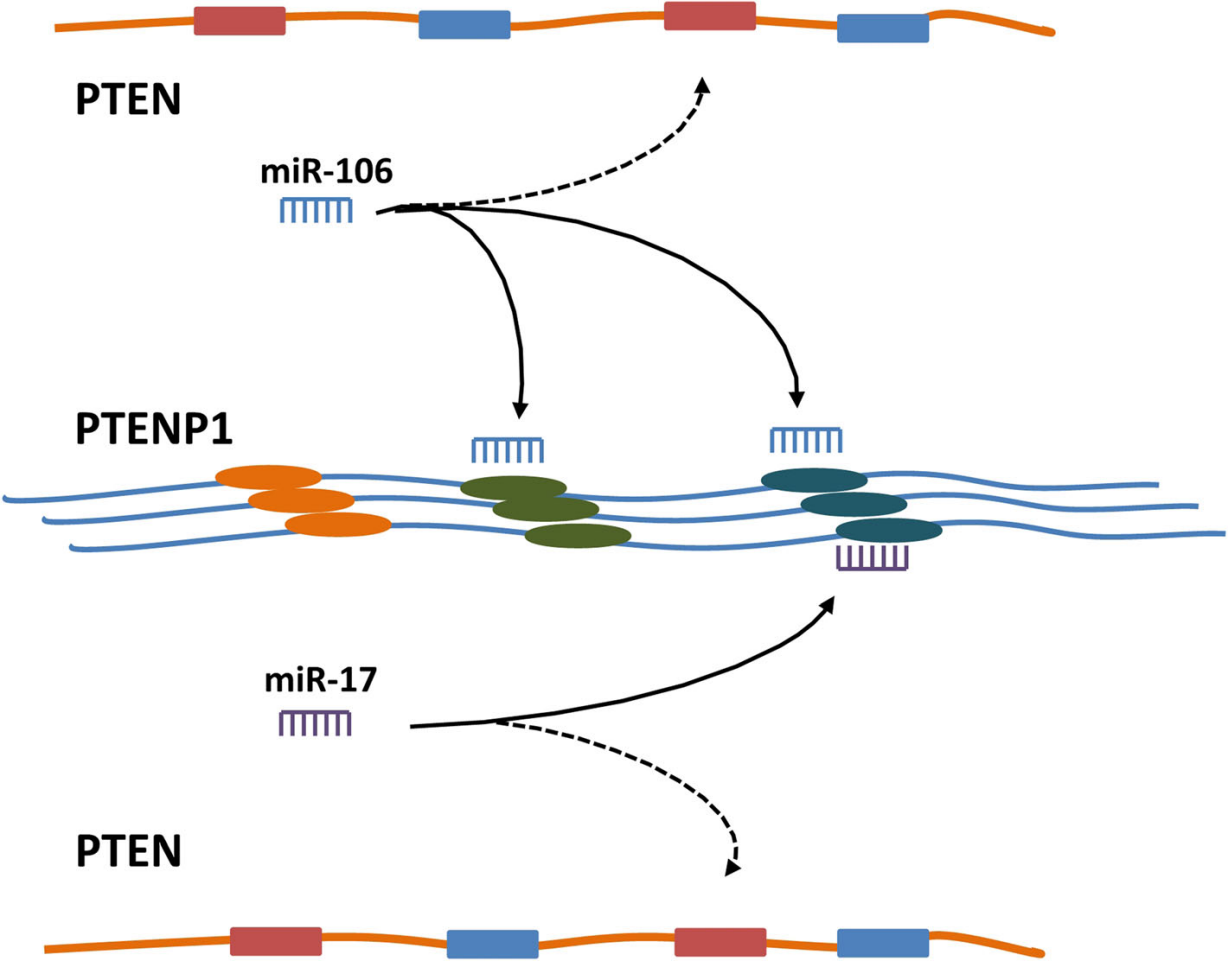
PTENP1 works as a ceRNA to promote PTEN expression. PTENP1 recruits miRNAs such as miR-181a and miR-21, therefore impairs the miRNAs binding PTEN

*PTENP1*, located on chromosome 9p21, is a highly conserved pseudogene of *PTEN*. Gan Yu et al. reported the low expression of PTENP1 due to methylation in CCRCC tissues and cell lines. Both PTEN and PTENP1 expression is inversely correlated with miR-21 expression. In miR-21 overexpressing cells, PTENP1 introduction suppressed cell proliferation and metastasis, and increased the cell sensitivity to cisplatin and gemcitabine, restoring the phenotypes induced by PTEN in vitro and in vivo [54]. Activation of PTENP1 partially inhibited the suppression of PTEN by miR-21 in oral squamous cell carcinoma (OSCC) tumor xenografts [96]. Evidences have revealed that PTENP1 expression level is positively related to PTEN transcript, and PTENP1 protects PTEN mRNA through serving as a decoy for miRNAs, such as miR-21, miR-17, miR-214, miR-19, miR-20, miR-93, miR-106b and miR-26 families [5, 54, 97–101] (Fig. 6).

Homeobox (HOX) transcript antisense RNA (HOTAIR) is encoded within the *HoxC* gene cluster on chromosome 12, which silences the expression of *HoxD* genes and numerous tumor and metastasis suppressors [102, 103] by interacting with chromatin-remodeling enzymes [104]. On the contrary, HOTAIR regulates PTEN expression as a ceRNA. HOTAIR expression decreased notably in sustained cardiac hypertrophy mouse models, in which miR-19 expression was increased and inversely correlated with HOTAIR expression. HOTAIR has a binding site for miR-19 seed sequence, and HOTAIR overexpression restored the inhibition of luciferase activity with PTEN 3’UTR mediated by miR-19 [105].

Linc-USP16 acted as a ceRNA for miR-21 and miR-590-5p, promoting PTEN expression to repress the growth and stimulate apoptosis in HCC in vivo and in vitro [106]. LncRNA-BGL3 worked as a ceRNA for miR-17, miR-93, miR-20a, miR-20b, miR-106a and miR-106b, rescuing the repression of PTEN expression to inhibit Bcr-Abl-induced cellular transformation [80].

### Acting as both ceRNAs and downregulators of miRNAs

LncRNAs can also decrease the expression level of miRNA as well as being sponges, leading to suppress the effects of miRNAs on their mRNA targets.

*Cancer susceptibility candidate 2 (CASC2)*, mapped to chromosome 10q26, encodes a lncRNA which acts as a ceRNA of miR-21 or miR-181a and exerts biological effects by increasing the expression of PTEN [107, 108]. The expression of CASC2 is significantly downregulated in glioma, osteosarcoma or cervical cancer tissues and cell lines, and CASC2 expression level is negatively correlated to miR-181a level in glioma tissues. CASC2 overexpression significantly repressed cell proliferation, and amplified temozolomide- or cisplatin-induced repression of cell proliferation in vitro, which was associated with the downregulation of miR-181a and miR-21. CASC2 overexpression upregulated PTEN level, which was partially restored by miR-181a and miR-21 mimics. In addition, CASC2 was found to interact directly with miR-181a and miR-21 in dual-luciferase reporter assays [108, 109].

*Maternally expressed gene 3 (MEG3)*, encoding a lncRNA, is located at the chromosome 14q32. In testicular germ cell tumor (TGCT) tissues, lncRNA MEG3 level is significantly decreased, while PTEN protein but not mRNA levels are notably downregulated [110]. Bioinformatics analyses showed that miR-1297 bound not only 3’UTR of PTEN mRNA but also MEG3 [111]. MEG3 overexpression disturbed the binding of miR-1297 to 3’UTR of PTEN mRNA and remitted the reduction of PTEN induced by miR-1297. MEG3 downregulation and miR-19a upregulation were reported in malignant glioma tissues and cell lines, and luciferase results verified the complementary binding between miR-19a and MEG3. MiR-19a overexpression repressed the expression of PTEN and promoted glioma cell proliferation, migration, and invasion [112]. Moreover, in the coronary artery disease (CAD) tissues, MEG3 level declines, and miR-21 expression has negative correlation with MEG3 expression. Overexpression of MEG3 suppressed miR-21 expression, promoted the expression of PTEN, and suppressed the proliferation of endothelial cells [113].

LncRNA growth arrest specific transcript 5 (lncRNA GAS5) is downregulated in NSCLC, breast cancer and HCC tissues, and lncRNA GAS5 knockdown repressed cell viability. lncRNA GAS5 competes with PTEN to bind miR-21, and depletion or overexpression of lncRNA GAS5 could increase or decrease miR-21 expression, resulting in the downregulation or upregulation of PTEN level in these tumor cells [114–116]. A low expression of lncRNA GAS5 and an upregulation of miR-21 are reported in cardiac fibrosis. The downregulation of PTEN expression mediated by miR-21 mimics was reversed by overexpressing lncRNA GAS5 in cardiac fibroblast cells [117]. LncRNA GAS5 could also induce PTEN expression by inhibiting miR-103 [118], miR-196a and miR-205 [119], and miR-32-5p [120].

The lncRNA X inactivate-specific transcript (XIST) directly interacts with miR-181a, and they repress the expression of each other. XIST overexpression restored the PTEN downregulation induced by miR-181a mimics, and transfection with XIST siRNA significantly enhanced the proliferation and invasion of liver cancer cells together with a decreased PTEN level [121]. Neuronal apoptosis and lncRNA XIST expression level were found to be promoted in an spinal cord injury model. XIST acts as a sink for miR-494, leading to derepression of PTEN. MiR-494 expression was upregulated with XIST knockdown, whereas was downregulated with XIST overexpression. AntagomiR-494 treatment reversed the protective effects of XIST depletion on spinal cord injury through blocking the PTEN/PI3K/AKT signaling pathway [122].

The low expression of LncRNA neuroblastoma associated transcript 1 (NBAT1) in osteosarcoma tissues and cells was closely correlated to clinical stages, lymph node metastasis and poor prognosis [123]. NBAT1 binds miR-21, and suppresses miR-21 expression. NBAT1 overexpression downregulated osteosarcoma growth and metastasis via acting as a ceRNA against miR-21, which was associated with PTEN upregulation in vitro and in vivo.

The expression of lnc-2 and lnc-6 showed positive correlation with PTEN in prostate cancer cohorts [124, 125]. Knockdown of lnc-2 or lnc-6 led to a significant decrease in PTEN expression at both protein and mRNA levels and a significant increase in cell proliferation. On the contrary, depletion of PTEN reduced the expression of both lnc-2 and lnc-6,and the reduction of PTEN expression by overexpressing known PTEN-regulating miRNAs could be rescued by overexpressing lnc-2 sub-sequences [126]. PTEN and lnc-6 are downregulated while miR-26a is upregulated in human glioma. Lnc-6 introduction into glioma cells resulted in a decrease of miR-26a expression [127].

Microarray and real time PCR results showed that lncRNA fer-1-like family member 4 (FER1L4) was downregulated in gastric cancer, endometrial carcinoma and colon cancer tissues or cell lines [128]. Enforced expression of FER1L4 increased PTEN expression at both mRNA and protein levels, which might contribute to cell cycle arrest and apoptosis [129]. In colon cancer cell lines, FER1L4 expression is inversely correlated with miR-106a-5p expression [130]. Luciferase assay results suggested direct interactions between miR-106a-5p and FER1L4 or PTEN. Knockdown of FER1L4 increased miR-106a-5p expression level and decreased the levels of PTEN mRNA and protein [130, 131].

Fujun Yu et al. reported a novel lincRNA-p21-miR-181b-PTEN signaling cascade in liver fibrosis [132]. LincRNA-p21 overexpression significantly suppressed the isolated rat HSC activation and the expression of extracellular matrix (ECM) proteins, which was reversed by depletion of PTEN. MiR-181b binds lincRNA-p21, and miR-181b level was reduced by exogenous lincRNA-p21, while the effects of lincRNA-p21 on PTEN expression and HSC activation were inhibited by miR-181b mimics.

### Increasing the stability of lncRNAs

*PTENP1*, also encodes antisense RNAs (asRNAs), which has two isoforms, α and β. PTENP1 asRNA β interacts with PTENP1 through an RNA:RNA pairing interaction, and the stability of PTENP1 was decreased when the interaction was interfering using U6 encoded ssRNAs or PTENP1 asRNA β was knocked down. Thus PTENP1 asRNA β upregualtes PTEN level via increasing the stability and miRNA sponge activity of PTENP1 [133].

### Prompting the methylation of PTEN promoter

HOTAIR expression is upregulated in HSCs during liver fibrosis. HOTAIR knockdown suppressed HSC proliferation and activation in vitro and in vivo, increasing PTEN level, with the loss of DNA methylation mediated by miR-29b [134]. HOTAIR levels were significantly higher in human laryngeal squamous cell cancer (LSCC), and bisulfite sequencing of the PTEN promoter addressed that PTEN CpG islands were unmethylated in HOTAIR siRNA-transduced cells and PTEN methylation was significantly reduced [135]. Collectively, HOTAIR might contribute to PTEN promoter methylation via sponging miR-29b.

The expression of PTEN and PTENP1 asRNA α is negatively correlated in cell lines, and the α depletion resulted in the increase of PTEN transcript. PTENP1 asRNA α binds the PTEN promoter, and epigenetically downregulates PTEN transcription by the recruitment of DNMT3a and Enhancer of zeste homolog 2 (EZH2) to enhance the methylation of PTEN promoter. PTENP1 asRNA α knockdown induces cell cycle arrest and sensitizes cells to doxorubicin, suggesting the biological function for the PTENP1 asRNAs [133, 136].

## Conclusions and future directions

Due to the essential physiological function of PTEN, the ncRNAs controlling PTEN expression play crucial roles in various biological activation, such as autophagy and cell stemness. PTEN induces autophagy through repressing PI3K/Akt pathway, while miR-21 elevation was found in human degenerative nucleus pulposus tissues, which inhibits autophagy and induces ECM degradation via repressing PTEN expression [137]; Human aortic smooth muscle cell-derived exosomal miR-221/222 suppressed the autophagy in human umbilical vein endothelial cells by regulating PTEN/Akt signaling pathway in a co-culture system [138]; MiR-21-5p significantly increases cell stemness of keloid keratinocytes, mediated by PTEN repression and AKT activation, which may account for the invasion and recurrence of keloids [139]. MiR-10b promotes cellular self-renewal and expression of stemness markers in breast cancer stem cells through negative regulation of PTEN and sustained activation of AKT [140].

Actually, therapeutic strategies for multiple diseases focus on PI3K/Akt pathway inhibitors. However, the therapeutic benefit is modest due to the network complexities [141, 142]. PTEN modulation has been considered as a possible approach to tumor and other diseases. NcRNAs including lncRNAs and miRNAs act alone or interact with each other to regulate PTEN expression. Elucidation of the details that ncRNAs modulate PTEN expression may provide novel insights into the regulation network of PTEN, which could suggest possible strategies to target PI3K/Akt pathway.

Primary therapeutic attempts targeting ncRNAs to alter the PTEN expression have shown effects. Sophocarpine, a tetracyclic quinolizidine alkaloid derived from *Sophora alopecuroides L*, has shown inhibitory effects on HNSCC progression via the downregulation of miR-21 and the upregulation of PTEN in vivo and in vitro [53]. Ursolic acid exerted protective action on high glucose-induced cell podocyte injury via decreasing miR-21 expression, which resulted in an increase of PTEN level [143]. Combination of STAT3 inhibitor and DDP treatment led to a notable reduction of STAT3/miR-21 axis and an increase of PTEN level, repressing oral squamous cell carcinoma (OSCC) cell proliferation, migration and invasion [144].

As-miR-21 treatment presented an obvious inhibition on established glioma tumor growth and an increase in PTEN expresson. Coincidently, in a prostate xenograft model, injection of as-miR-4534 led to a significant reduction in tumor volume, which increased the expression level of PTEN [145]. In a spontaneously developed lung tumors mouse model, treatment with the miR-214 antisense oligonucleotides microvesicles displayed promotion of PTEN levels and reduction of growth of spontaneous lung tumors [68]. Furthermore, administration of LNA-antimiR-19a increased the sensitivity of multidrug resistant MCF-7 cells to Taxol in vivo, with an upregulation of PTEN verified [146]. The growth of human LSCC xenograft was remarkably inhibited by HOTAIR shRNA lentivirus treatment [135], and injection of the PTENP1-expressing baculovirus effectively mitigated the HCC xenograft tumor growth, which was associated with the increase of PTEN [97].

In term of the importance of PTEN expression level in physiological situation and pathogenesis of various diseases, modulating PTEN level could be considered as potential approaches for multiple diseases, while clarifying the regulation network of PTEN including ncRNAs is predicted to be able to provide novel strategies.

## Abbreviations

AML: Myeloid leukemia
C2D: C2 membrane binding domain
CAD: Coronary artery disease
CASC2: Cancer susceptibility candidate 2
CCRCC: Clear-cell renal cell carcinoma
ceRNAs: Competing endogenous RNAs
CK2: Formerly casein kinase II
CSCs: Cancer stem cells
DNMTs: DNA methyltransferases
ECM: Extracellular matrix
EMT: Mesenchymal transition
eNOS: Endothelial nitric oxide synthase (eNOS)
ERK1/2: Extracellular signal-regulated kinase
FAK: Focal adhesion kinase
FER1L4: lncRNA fer-1-like family member 4
GSK3: Glycogen synthase kinase3
HCAECs: Human coronary artery endothelial cells
HNSCC: Head and neck squamous cell carcinoma
HOTAIR: Homeobox (HOX) transcript antisense RNA
HSC: Hepatic stellate cell
lncRNA GAS5: lncRNA growth arrest specific transcript 5
lncRNAs: Long ncRNAs
LSCC: Laryngeal squamous cell cancer
mDA: Midbrain dopaminergic
Meg3: Maternally expressed gene 3
miRNAs: Small ncRNAs
MMAC1: Mutated in multiple advanced cancers
mTORC: Mammalian target of rapamycin complex
NBAT1: LncRNA neuroblastoma associated transcript 1
ncRNAs: Noncoding RNAs
NSCLC: Non-small cell lung cancer
OSCC: Oral squamous cell carcinoma
PBD: Phosphatidylinositol-4,5-bisphosphate-binding domain
PD: A catalytic domain phosphatase domain
PD: Parkinson’s disease
PDZ: Post-synaptic density protein (PSD95), Drosophila discs large (Dlg) and the tight junction protein zonula occludens-1 (ZO-1)
PDZK1: PDZ-containing 1
PI3K: Phosphatidylinositol 3-kinase
PIP3: Phosphatidylinositol (3,4,5)-triphosphate
PTEN: Phosphatase and tensin homologue
PTENP1: Pseudogene of PTEN
SET1A: SET domain containing 1A
Shc: Src homology 2-containing protein
TGCT: Testicular germ cell tumor
TRAIL: TNF-related apoptosis-inducing ligand
TSC2: Tuberous Sclerosis Complex 2
XIST: lncRNA X inactivate-specific transcript
